# Development and validation of a diagnostic model for migraine without aura in inpatients

**DOI:** 10.3389/fneur.2025.1511252

**Published:** 2025-01-21

**Authors:** Zhu-Hong Chen, Guan Yang, Chi Zhang, Dan Su, Yu-Ting Li, Yu-Xuan Shang, Wei Zhang, Wen Wang

**Affiliations:** ^1^Functional and Molecular Imaging Key Lab of Shaanxi Province, Department of Radiology, Tangdu Hospital, Air Force Medical University, Xi’an, Shaanxi, China; ^2^Department of Medical Imaging, Gansu Corps Hospital of Chinese Armed Police Force, Lanzhou, Gansu, China; ^3^Department of Medical and Technology, Shaanxi University of Chinese Medicine, Xianyang, Shaanxi, China; ^4^Department of Neurology, Tangdu Hospital, Air Force Medical University, Xi’an, Shaanxi, China

**Keywords:** migraine, migraine without aura, logistic regression model, diagnosis, patent foramen ovale

## Abstract

**Objectives:**

This study aimed to develop and validate a robust predictive model for accurately identifying migraine without aura (MWoA) individuals from migraine patients.

**Methods:**

We recruited 637 migraine patients, randomizing them into training and validation cohorts. Participant’s medical data were collected such as demographic data (age, gender, self-reported headache characteristics) and clinical details including symptoms, triggers, and comorbidities. The model stability, which was developed using multivariable logistic regression, was tested by the internal validation cohort. Model efficacy was evaluated using the area under the receiver operating characteristic curve (AUC), alongside with nomogram, calibration curve, and decision curve analysis (DCA).

**Results:**

The study included 477 females (average age 46.62 ± 15.64) and 160 males (average age 39.78 ± 19.53). A total of 397 individuals met the criteria for MWoA. Key predictors in the regression model included patent foramen ovale (PFO) (*OR* = 2.30, *p* = 0.01), blurred vision (*OR* = 0.40, *p* = 0.001), dizziness (*OR* = 0.16, *p* < 0.01), and anxiety/depression (*OR* = 0.41, *p* = 0.02). Common symptoms like nausea (*OR* = 0.79, *p* = 0.43) and vomiting (*OR* = 0.64, *p* = 0.17) were not statistically significant predictors for MWoA. The AUC values were 79.1% and 82.8% in the training and validation cohorts, respectively, with good calibration in both.

**Conclusion:**

The predictive model developed and validated in this study demonstrates significant efficacy in identifying MWoA. Our findings highlight PFO as a potential key risk factor, underscoring its importance for early prevention, screening, and diagnosis of MWoA.

## Strengths and limitations of this study

Mitigated recall bias through medical records;

Established a predictive model for MWoA;

Disclosed the relationship between MWoA and PFO;

A broader scope of study needs to be expanded in the future.

## 1 Introduction

Migraine, one of the most prevalent conditions of primary headache, affects approximately 15% of the global population, with a higher prevalence among women than men (1). For Chinese individuals, the overall prevalence was approximately 10% (2). Furthermore, the incidence among women in China has been consistently increasing and is projected to continue this trend till 2030 (3).

Beyond recurrent episodes of moderate to severe headaches, migraine was frequently accompanied by a host of concomitant symptoms, including nausea, vomiting, photophobia, and phonophobia, and a range of comorbidity symptoms, such as anxiety, depression, patent foramen ovale (PFO), hypertension, cerebrovascular disease, etc. (4).

Previous research has identified various demographic, clinical, and genetic factors contributing to the risk of developing migraine (5). Gender has been consistently identified as a significant factor in migraine prevalence, with women being more likely to experience migraine than men (1). Age has also been shown to influence the onset and frequency of migraine, with peak prevalence occurring in individuals aged 25–50 years (6). Additionally, genetic factors have been implicated in the pathogenesis of migraine, with a family history of migraine increasing the risk of developing the condition (7).

Given these clinical characteristics, identifying predictive factors in migraine is crucial for developing personalized medicine plans and implementing targeted interventions. Moreover, clinical factors can predict the occurrence, severity, and response to treatment of migraine individuals (8).

Clinically, however, the headache characteristics of migraine (like frequency, severity, and headache location) varied from month to month. The triggers for migraine attacks (like menstrual cycle, caffeine, alcoholic drinks, stress, tension, and fatigue) were often acknowledged as general and nonspecific triggers of headache (9, 10). The available data indicates that Chinese migraine patients more commonly present with nausea and vomiting (11), whereas Caucasian patients often exhibit photophobia and phonophobia (12).

Unlike migraine patients with aura, which was characterized by distinct aura symptoms, migraine without aura (MWoA) patients lack such clear precursors, making its distinction from other migraine subtypes challenging. There is no existing predictive model with both sensitively and specifically based on headache features (attack frequency, duration), symptoms, and headache-related disability (13) to reliably differentiate between migraine with or without aura from individual sufferers (14).

Understanding these clinical factors and their interactions can help clinicians identify high-risk migraine patients and tailor treatment plans accordingly. The symptoms (nausea, photophobia, phonophobia, etc.) of migraine could be given a better model predictor to assess medication usefulness (15). Furthermore, migraine with anxiety and depression might decrease the likelihood of response to (nonsteroidal anti-inflammatory drugs) NSAIDs, and play a critical role in predicting the treatment outcomes of acute migraine (16).

MWoA was the most common type among the spectrum of migraine disorders (2). In the current study, we hope to develop a predictive model of MWoA and other migraine patients through clinical characteristics. This can help us accurately identify and initially intervene to prevent MWoA attacks.

## 2 Methods

### 2.1 Participants

This study was approved by the Ethics Committee of Tangdu Hospital (TDLL-2015133). Informed consent was not required for this study as it exclusively relied on the analysis of observational data obtained from the patient information administration and registration system. Totally 736 patients diagnosed with migraine were retrieved, from January 2002 to January 2023. The diagnosis was made based on the International Classification of Headache Disorders, 3rd edition (ICHD-3).

The inclusion criteria: patients with a confirmed diagnosis of migraine (including recurrent headache attacks lasting 4–72 h; headache is unilateral location; pulsating quality; moderate or severe pain intensity; aggravation by or causing avoidance of routine physical activity; association with nausea and/or photophobia and phonophobia); patients with available data on gender, age, trigger factor, clinical symptoms, comorbidity, and other relevant suffering performance. Exclusion criteria include: other primary headache disorders (e.g., cluster headache, tension-type headache, medication-overuse headache, post-traumatic headache, etc.), neurological diseases, psychiatric disorders, and rheumatic diseases.

A total of 99 individuals were excluded, among whom 27 were due to repeated admissions, 26 due to incompleted medical history records, and 46 because migraine was listed as a comorbid diagnosis (12 participants for malignant/metastatic tumor, 26 participants for cerebral hemorrhage sequelae, and 8 participants for medication overuse headache).

### 2.2 Data collection

Ultimately, 637 patients were included in this analysis. The following demographic data and headache characteristics were obtained: age, gender, headache location (including left, right, bilateral side, or others), and the nature of pain (pulsating, bursting, stabbing, or others).

Moreover, clinical characteristics were collected: prodromal symptoms including blurred vision and dizziness, trigger factors such as mood changes, fatigue, influenza, and menstruation. Suffering symptoms, photophobia, phonophobia, nausea, and vomiting, were also included. All those symptoms were self-reported by the migraine patients and confirmed during consultation with a neurologist. The comorbidities included hypertension, type 2 diabetes mellitus (diabetes), patent foramen ovale (PFO), anxiety/depression, and cerebrovascular disease (including lacuna infarction, white matter hyperintensities, and lacunar ischemic stroke). The anxiety/depression levels of the patients were assessed by the Self-rating Depression Scales (SDS) (17) and Self-rating Anxiety Scales (SAS) (18). PFO was diagnosed using contrast echocardiography.

### 2.3 Statistical analysis

The dataset of migraine patients was randomly divided into training and validation cohorts at a ratio of 7:3, and the variables were compared. Normally data were listed as mean ± standard deviation (SD), while non-normal data were presented as median (interquartile ranges). In the univariate analysis, the chi-square test or Fisher’s exact test was used to analyze the categorical variables. Comparisons were using the Student’s *t*-test or Mann–Whitney *U* test to examine the continuous variables. To reduce the effect of multicollinearity on the regression results, the least absolute shrinkage and selection operator (LASSO) logistic regression (19) analysis was used for multivariate analysis to screen the independent risk factors, and the logistic regression model was used to establish a predictive nomogram for MWoA in the training cohort. The performance of the nomogram was assessed using the receiver operating characteristic (ROC) curve, with the area under curve (AUC) ranging from 0.5 (no discriminant) to 1 (complete discriminant). The Hosmer-Lemeshow goodness of fit test in the multiple logistic regression was performed to assess the model calibration and the calibration plot was plotted. A decision curve analysis (DCA) was also performed to determine the net benefit threshold of prediction. There were missing data for patients’ headache characteristics (like headache location, pain nature), triggers for headache attacks, and comorbidities. In the logistic regression model, only the subjects with complete data in all variables were considered. The multiple imputations were used to assess the sensitivity of results to missing values (20). Results with a *p* value of <0.05 were considered significant. All statistical analyses were performed using the R software (Version 4.2.2).

## 3 Results

### 3.1 Demographic characteristics

Of the included 637 migraine patients, 477 (46.62 ± 15.64) were females and 160 (39.78 ± 19.53) were males. There were no significant differences observed in the distribution of different age groups among the training test cohort (*N* = 446) and internal validation test cohort (*N* = 191) (Table 1).

**Table 1:** Patient demographics and baseline characteristics for training and validation cohort.

Variables	Training cohort *N* = 446	Validation cohort *N* = 191	*P* ^*^
Age			0.91
0–17 years	39 (8.7%)	20 (10.5%)	
18–44 years	153 (34.3%)	64 (33.5%)	
45–65 years	209 (46.9%)	87 (45.5%)	
65 + years	45 (10.1%)	20 (10.5%)	
Gender			0.84
Male	111 (24.9%)	49 (25.7%)	
Female	335 (75.1%)	142 (74.3%)	
Location			0.59
Left	116 (26.0%)	41 (21.5%)	
Right	89 (20.0%)	44 (23.0%)	
Bilateral	164 (36.8%)	70 (36.6%)	
Others	77 (17.3%)	36 (18.8%)	
Nature			0.34
Throbbing	172 (38.6%)	68 (35.6%)	
Bursting	117 (26.2%)	63 (33.0%)	
Stabbing	37 (8.3%)	12 (6.3%)	
Others	120 (26.9%)	48 (25.1%)	
Mood	28 (6.3%)	20 (10.5%)	0.07
Fatigue	61 (13.7%)	33 (17.3%)	0.24
Influenza	40 (9.0%)	11 (5.8%)	0.17
Blurred vision	94 (21.1%)	36 (18.8%)	0.52
Dizziness	156 (35.0%)	66 (34.6%)	0.92
Photophobia	59 (13.2%)	23 (12.0%)	0.68
Phonophobia	40 (9.0%)	15 (7.9%)	0.65
Nausea	216 (48.4%)	96 (50.3%)	0.67
Vomiting	133 (29.8%)	71 (37.2%)	0.07
Hypertension	99 (22.2%)	40 (20.9%)	0.73
Diabetes	26 (5.8%)	13 (6.8%)	0.64
PFO	85 (19.1%)	29 (15.2%)	0.24
Anxiety/Depression	43 (9.6%)	16 (8.4%)	0.61
Cerebrovascular	87 (19.5%)	33 (17.3%)	0.51

^*^Pearson’s Chi-squared test. All comparisons were statistically non-significant.

The majority age group of participants was the 45–65 years category, representing 46.5% of the overall individuals, with a similar proportion in the two test cohorts. The females comprised approximately 74.9% of the total sample, and the distribution also remained across cohorts.

The attacking headache of migraine was located in bilateral (36.73%) with throbbing nature (37.68%), a similar pattern across cohorts. Fatigue (14.76%) emerged as the predominant trigger factor. The higher proportion of prodromal symptoms were dizziness (34.85%) and blurred vision (20.41%). Nausea (48.80%) and vomiting (32.03%), although commonly suffered symptoms of migraine, and no substantial significant difference across cohorts was detected (Table 1).

The prevalence of comorbid conditions such as PFO, hypertension, diabetes, and cerebrovascular disease was consistent among the cohorts, with no significant differences. Overall, the baseline characteristics of the study population were largely consistent across cohorts, providing a solid foundation for further predictive analyses (Table 1).

There was no significant variance inflation factor (VIF) among variables, there were no outliers, and the included variables met the linearity assumption (Supplementary Table S2). Multiple imputations for missing data generated similar results.

### 3.2 Predictive model

**Figure 1:**
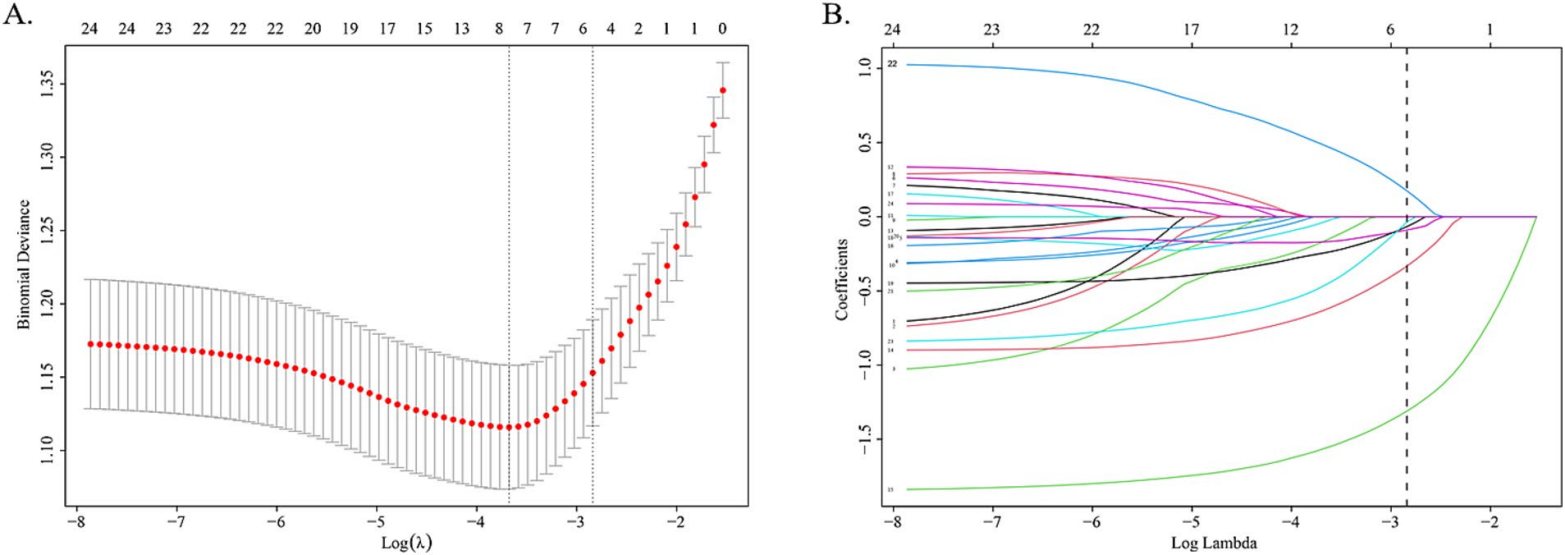
LASSO regression analysis gram. **(A)** Cross-validation plot of LASSO regression. The value in the middle of the two dotted lines is the range of the positive and negative standard deviations of the log(*λ*). The dotted line on the left indicates the value of the harmonic parameter log(λ) when the error of the model is minimized. Six variables were screened when log(λ) = −2.57. **(B)** LASSO coefficient profiles of the 24 variables. A vertical line was drawn at the value chosen by 10-fold cross-validation. As the value of λ decreased, the degree of model compression increased and the function of the model to select important variables increased.

Using LASSO regression analysis performed in the training cohort, 6 potential predictors were included from the candidate variables in the original model, Age, Gender, Location of headache, Headache nature, Mood changes, Fatigue, Influenza, Blurred vision, Dizziness, Photophobia, Phonophobia, Nausea, Vomiting, Hypertension, Diabetes, PFO, Anxiety/Depression, and Cerebrovascular disease (Figure 1).

**Figure 2:**
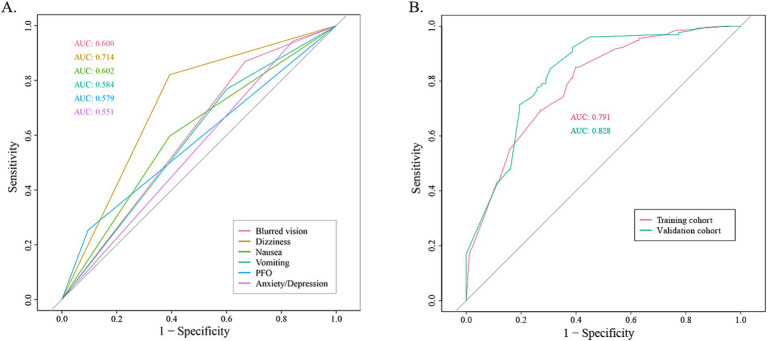
The receiver operating characteristic curve (ROC). **(A)** The ROC for each recruited variable. **(B)** The ROC for training cohort and validation cohort, respectively.

The coefficients of included predictors were estimated, blurred vision (−0.33), dizziness (−1.31), nausea (−0.09), vomiting (−0.07), PFO (0.17), and anxiety/depression (−0.03). A cross-validated error plot of the LASSO regression model was also exhibited. The most regularized and parsimonious model, with a cross-validated error within one standard error of the minimum, conclusively included 6 variables. The receiver operating characteristic curves (ROC) were yielded for the abovementioned variables’ area under curve (AUC) values greater than 0.5 and the prediction model was derived from the multivariate logistic regression for training and validation cohort (Figure 2; Supplementary Table S1).

**Figure 3:**
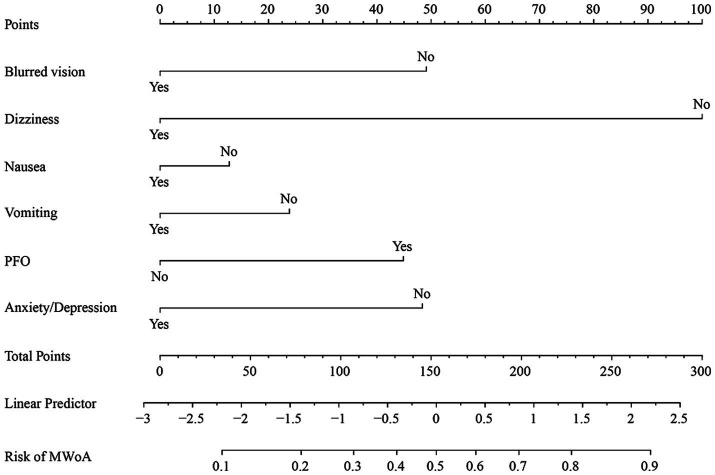
Nomogram of training cohort for MWoA probability. The axis of each variable was assigned a plotted score for a migraine patient, and these scores were summed and plotted on the line to obtain the total point score. The total point score corresponds to the predicted probability of MWoA.

**Figure 4:**
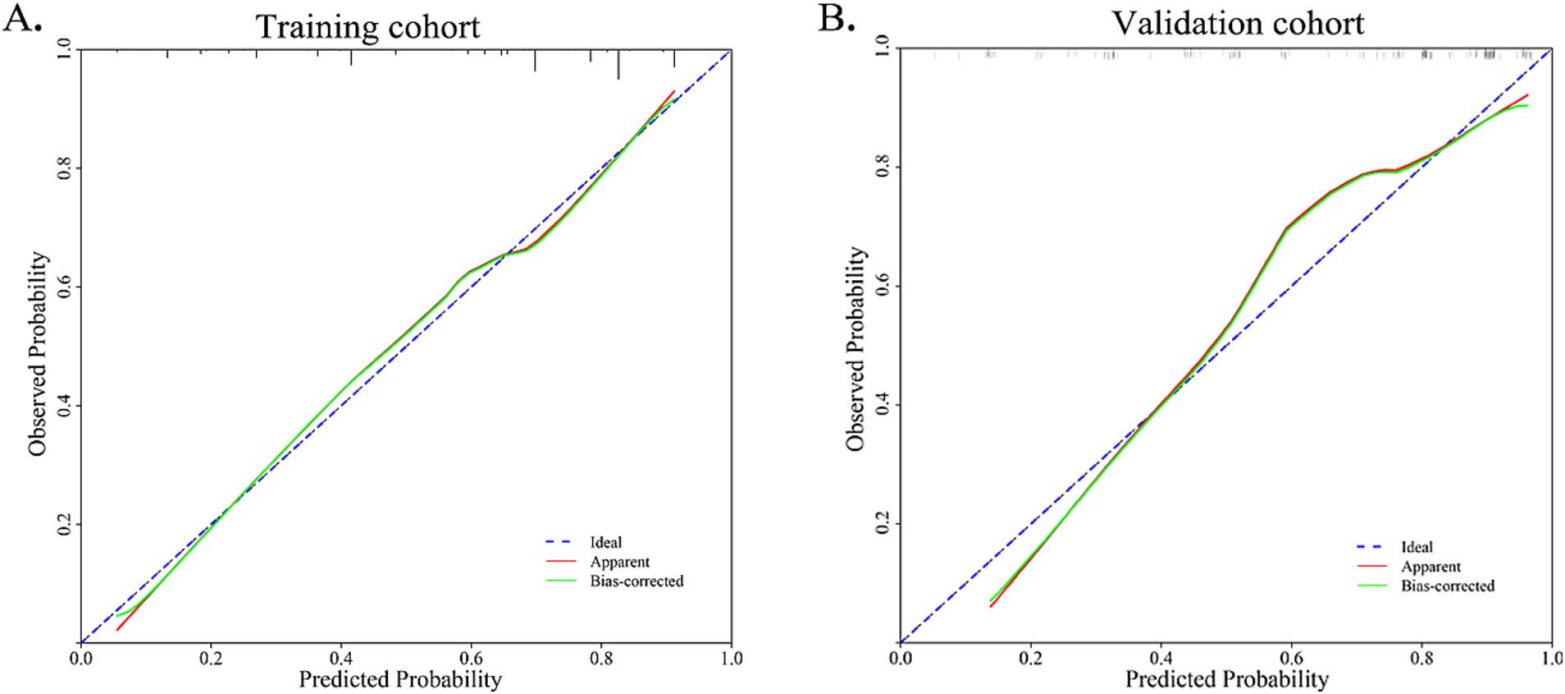
Calibration curve for training cohort and validation cohort. The Hosmer-Lemeshow goodness fit showed a *p*-value of 0.598 for the training cohort and 0.179 for the validation cohort.

The nomogram and calibration plots among the different cohorts were plotted, which demonstrated a good correlation between the observed and predicted migraine subtype (Figure 3). Findings indicated that the initial nomogram remained applicable for the validation sets, with the calibration curve of the model closely approximating the ideal curve (Figure 4). The Hosmer-Lemeshow goodness fit showed a *p*-value of 0.598 for the training cohort and 0.179 for the validation cohort. These results suggest a high level of consistency between predicted outcomes and actual observations.

Nomogram for predicting the probability of MWoA. The presence or absence of each clinical characteristic indicates a certain number of points. For each characteristic, absence is assigned 0 points. The presence of characteristic is generated using R based on the results of LASSO analysis. The points for each characteristic are summed together to generate a total points score.

Further multivariate logistic analyses were carried out in the training cohort. We found that PFO (*OR* = 2.30, *p* = 0.01), blurred vision (*OR* = 0.40, *p* = 0.001), dizziness (*OR* = 0.16, *p* < 0.01), and anxiety/depression (*OR* = 0.41, *p* = 0.02) was the influencing factor for the MWoA. Symptoms of nausea (*OR* = 0.79, *p* = 0.43), and vomiting (*OR* = 0.64, *p* = 0.17) did not enhance the risk of the suffering of MWoA (Table 2).

**Table 2:** Results of multivariate logistic regression for training cohort.

Variables	*β*	SE	OR	95% CI		*P*
				Lower	Upper	
Blurred vision	−0.91	0.28	0.40	0.23	0.69	**0.001**
Dizziness	−1.85	0.23	0.16	0.10	0.25	**<0.001**
Nausea	−0.24	0.30	0.79	0.44	1.43	0.43
Vomiting	−0.44	0.32	0.64	0.34	1.20	0.17
PFO	0.83	0.33	2.30	1.22	4.52	**0.01**
Anxiety/Depression	−0.90	0.40	0.41	0.19	0.88	**0.02**

CI, confidence interval; OR, odds ratio; SE, standard error. Bolded *P*-values indicate statistical significance.

According to the diagnostic criteria of the ICHD-3, nausea and vomiting are essential symptoms for the diagnosis of migraine. Additionally, after removing these two variables, the AUC value of the model decreases (the AUC values for the training cohort and validation cohort were 0.773 and 0.826, respectively, Supplementary Figure S1). Meanwhile, these two variables exhibiting a high prevalence among migraine patients were included in the final model to intensify its clinical practical guidance significance.

Variables (such as blurred vision, dizziness, nausea, vomiting, and anxiety/depression) with a negative *β* value can be considered protective predictive factors for the diagnosis of MWoA, whereas PFO is a risk predictive factor for MWoA.

### 3.3 Decision curve analysis

**Figure 5:**
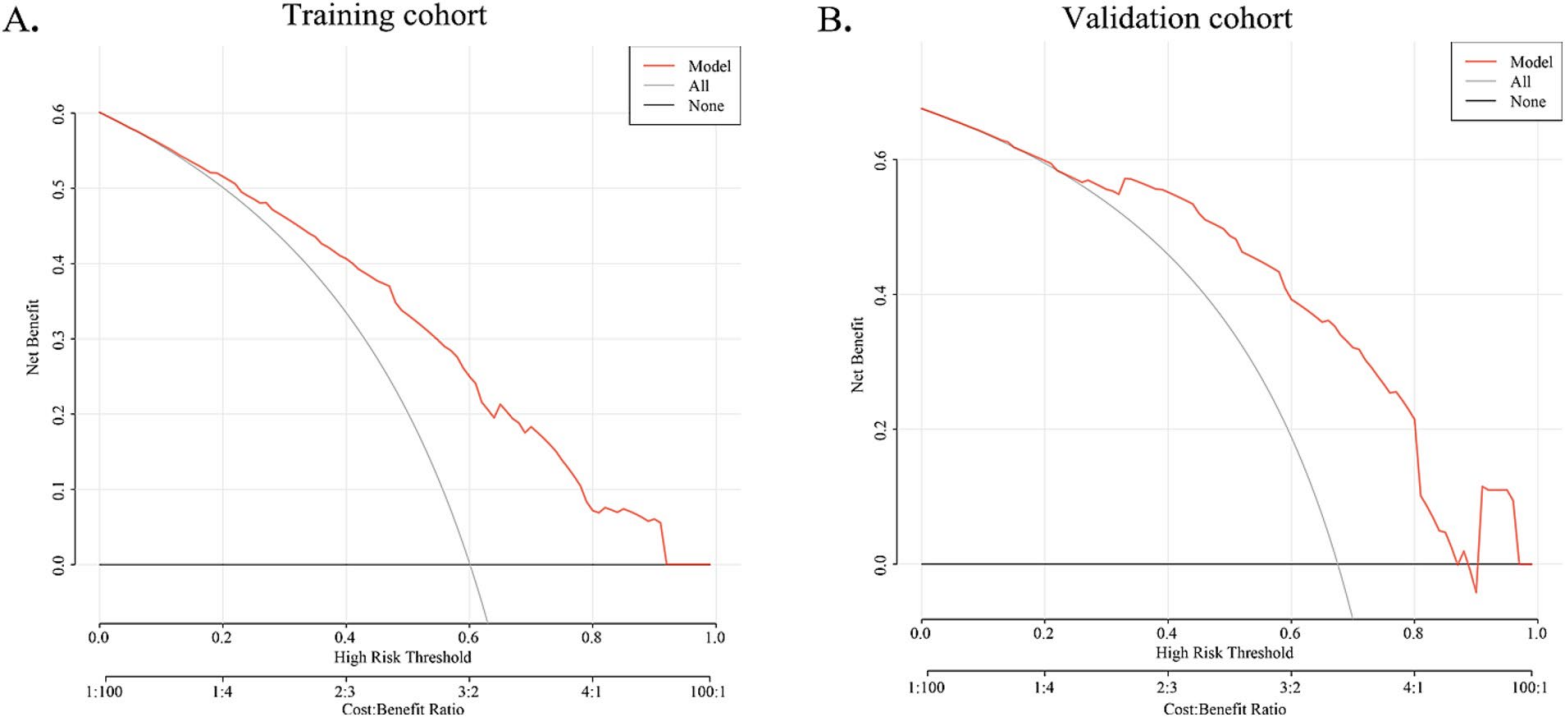
DCA curve for training cohort and validation cohort. The X-axis indicates the threshold probability for MWoA and the Y-axis shows the net benefit. The red line represents the predictive nomogram. The gray line represents the scenario where all patients are assumed to have MWoA, while the black line represents the scenario where no patients are assumed to have MWoA. In training and validation cohorts, this decision curve indicates that the application of this nomogram would provide a net benefit.

The DCA curve of the nomogram indicated the chance of substantial divergence in the predictive accuracy of the model when clinicians encounter imperfections during the utilization of the nomogram for diagnostic deliberation and the diagnostic decision-making processes. The present study demonstrated that the nomogram confers considerable advantages in terms of clinical utility, as evidenced by its favorable performance on the DCA curve (Figure 5).

## 4 Discussion

In this study, we developed and validated a nomogram to identify MWoA from migraine sufferers. The main predictors of the nomogram, like PFO, blurred vision, dizziness, and anxiety/depression, were statistically significant in multivariate logistic regression analysis.

Based on clinical manifestations, it has been reliably differentiated vestibular migraine (VM) and Meniere’s disease using attack frequency (21), phonophobia, nausea, vomiting, and dizziness (22). However, fewer studies focused on the attention between MWoA and other subtypes of migraine identification. Given MWoA the largest number of sufferers and no explicit aura symptoms, it is difficult to differentiate it from other primary headaches based on clinical characteristics merely, and the establishment of predictive models to analyze synthesized risk factors is clinically crucial for the early identification and intervention treatment.

### 4.1 The risk factors

The relationship between clinical characteristics and the risk of migraine was initially investigated in this study, and their predictive for MWoA was assessed subsequently. The results not only validated the predictive significance of PFO, blurred vision, dizziness, and anxiety/depression for MWoA, but also indicated a substantial association between PFO and MWoA, as well as a certain predictive capacity.

The PFO is a remnant of fetal circulation and is also the most common congenital cardiac anomaly in adult populations (23). Multiple studies suggest that migraine is more prevalent in subjects with PFO and vice versa (24). Several studies found that the incidence of PFO in migraine was 14.6–66.5% (25). In turn, in the population with PFO, the incidence of migraine was 9.13–51.7% (24). Some studies have shown that there is a stronger relationship between migraine with aura and PFO, the incidence of PFO is 46.3–88% in migraine patients with aura compared with 16.2–34.9% in migraine patients without aura (24). It has also been found that the atypical aura group was 79.2% vs.46.3% in the typical aura group (26). In China, a community-based cross-sectional study pointed to a strong association between PFO and MWoA, especially when the shunt is large (27). This difference might be due to racial genetic or regional disparities.

Therefore, we suggest that the pathophysiological relationship between PFO and aura in migraine patients needs to be further explored. However, the likelihood of benefit from PFO closure would appear to be increased, especially for migraine sufferers who have failed multiple pharmacological interventions (28), by cessation of migraine headaches or reducing migraine attacks and migraine days (29).

PFO can also serve as a significant sign for early classification and diagnosis of migraine patients. It can be considered that patients with PFO have a higher probability of suffering from migraine, and conversely, patients with migraine are more likely to have a PFO.

Anxiety/depression has a significant association with migraine allodynia (30), and migraine patients were more likely to be females who reported higher levels of current anxiety symptoms (31). Epidemiological studies have shown that patients with migraine were three times more likely to suffer from depression (1) and four to fivefold increase in the risk of anxiety (32) than the general population.

In recent studies, anxiety/depression has been confirmed a close correlation with migraine (33). Patients with migraine had worse depression and anxiety than those without (34). Depression and anxiety were the predictive factors of beliefs related to constancy of pain (35). Depression, headache features (higher headache pain intensity, more headache days per month), and no pharmacologic treatment factors (not using preventive migraine medications) were significant predictors of inadequate 2-h pain-free (36). There is a higher risk of transformation that migraine with anxiety/depression to chronic migraine, and risks are more pronounced in migraine with aura than in MWoA (2). Consistent with previous studies, this study demonstrated that MWoA had a lower risk of developing anxiety/depression than other patients.

The prodromal phase symptoms can be in some situations confused with migraine aura (37), and the description is not clear enough especially for the visual symptoms (38). It can be described as “foggy vision,” “dimness,” blurred vision for near or far objects, and blind or black spots. The prodromal symptoms of migraine, such as sensitivity to light or noise, neck stiffness, fatigue, and difficulty concentrating, had been reported to be prevalent in migraine patients through the studies of questionnaires, clinician interviews, diaries, or retrospective recall (39, 40). From 122 migraine with aura patients shown that the most visual symptom was blurred vision (41). Blurred vision accounted for 17.9% of participants of chronic migraine (42). Friedman and Evans have proposed that blurred vision may be a symptom of autonomic dysfunction due to an imbalance in sympathetic and parasympathetic signaling (43).

Along with dizziness, migraine was also characterized by symptoms such as phonophobia, motion intolerance, nausea, vomiting (44), and photophobia (45). In fact, up to 15.5% of visits to general healthcare settings were related to concerns about dizziness (46). Interestingly, in some migraine patients, the dizziness and vomiting can be more debilitating than the headache itself (47).

Meanwhile, MWoA had a lower risk of suffering prodromal symptoms like blurred vision, dizziness, and common symptoms of nausea and vomiting. This indicates, from one perspective, that patients with MWoA tend to have few other positive physical signs beyond their headache symptoms, necessitating heightened attention during clinical differential diagnosis. For migraine, early intervention may be the most effective acute treatment strategy (8). It also suggests that when treating and evaluating treatment efficacy, particular emphasis should be placed on the alleviation of headache symptoms.

### 4.2 The predictive model

This predictive model ultimately utilized PFO, blurred vision, dizziness, nausea, vomiting, and anxiety/depression to identify MWoA, which has a high ROC value. Meanwhile, when LASSO regression was used to select variables, variables such as gender, age, occupation, and the headache nature were excluded, making the model more convenient.

The AUC values for the training and validation cohort were 0.791 and 0.828, respectively, indicating that this nomogram has good accuracy and stability. The nomogram is relatively easy to use due to the fewer number of included variables, and the operation is also easy to master. This model can obtain a total score of variables and the probability of developing MWoA, thus helping clinicians provide more beneficial advice to patients. For example, if a migraine has a PFO and does not experience symptoms such as blurred vision, dizziness, nausea, vomiting, or anxiety/depression, their total score would be approximately 270, corresponding to a probability of about 90% for MWoA. The developed nomogram offers several clinical implications. Firstly, it provides a quantitative tool for clinicians to predict MWoA more accurately than traditional methods, aiding in better risk stratification for comorbidity PFO. Moreover, early identification of high-risk individuals through this nomogram can lead to timely interventions, potentially reducing morbidity and mortality.

### 4.3 Limitation

Our study has several limitations that should be acknowledged. The single-center retrospective study was based on patients from a hospital in Northwest China, which may not be representative of the wider population. Furthermore, there might be potential unmeasured confounders that were not included in our model. External validation in diverse populations will be essential to confirm the generalizability of our findings.

## 5 Conclusion

In conclusion, blurred vision, dizziness, vomiting, photophobia, phonophobia, and PFO can be considered clinical markers for classifying and monitoring migraine. Further studies with larger sample sizes in diverse populations are needed to confirm the discrepancy, serving as references for clinicians.

## Data Availability

The original contributions presented in the study are included in the article/Supplementary material, further inquiries can be directed to the corresponding authors.
