# Supplementary material for: Development and validation of a diagnostic model for migraine without aura in inpatients
Source: Front Neurol. 2025 Jan 21;16:1511252. doi: 10.3389/fneur.2025.1511252 (PMC11790451; doi:10.3389/fneur.2025.1511252)
Supplement: Supplementary file 1 [file Table_1.docx]

S-Table1. Classification accuracy for prediction at different risk cutoff points for the model in training cohort.

| Risk score threshold | Linear Predictor  Cutoff Point | Sensitivity (%) | Specificity (%) | PPV (%) | NPV (%) | Accuracy (%) | Precision (%) | Recall (%) |
| --- | --- | --- | --- | --- | --- | --- | --- | --- |
| ≥ 0% | -Inf | 100.0 | 0.0 | 60.1 |  | 60.1 | 60.1 | 100.0 |
| ≥ 10% | -2.1972246 | 100.0 | 4.5 | 61.2 | 100.0 | 61.9 | 61.2 | 100.0 |
| ≥ 20% | -1.3862944 | 98.5 | 23.6 | 66.0 | 91.3 | 68.6 | 66.0 | 98.5 |
| ≥ 30% | -0.8472979 | 94.8 | 37.1 | 69.4 | 82.5 | 71.7 | 69.4 | 94.8 |
| ≥ 40% | -0.4054651 | 91.8 | 45.5 | 71.7 | 78.6 | 73.3 | 71.7 | 91.8 |
| ≥ 50% | 0.0000000 | 81.0 | 61.2 | 75.9 | 68.1 | 73.1 | 75.9 | 81.0 |
| ≥ 60% | 0.4054651 | 78.0 | 63.5 | 76.3 | 65.7 | 72.2 | 76.3 | 78.0 |
| ≥ 70% | 0.8472979 | 54.9 | 84.3 | 84.0 | 55.4 | 66.6 | 84.0 | 54.9 |
| ≥ 80% | 1.3862944 | 44.8 | 87.6 | 84.5 | 51.3 | 61.9 | 84.5 | 44.8 |
| ≥ 90% | 2.1972246 | 16.8 | 98.9 | 95.7 | 44.1 | 49.6 | 95.7 | 16.8 |
| ≥ 100% | Inf | 0.0 | 100.0 |  | 39.9 | 39.9 |  | 0.0 |
