# Supplementary material for: Development and validation of a diagnostic model for migraine without aura in inpatients
Source: Front Neurol. 2025 Jan 21;16:1511252. doi: 10.3389/fneur.2025.1511252 (PMC11790451; doi:10.3389/fneur.2025.1511252)
Supplement: Supplementary file 2 [file Table_2.docx]

S-Table 2. The variance inflation factor and tolerance for variables.

| **Variables** | **VIF** | | **VIF（95%CI）** | | **Tolerance** | **Tolerance（95%CI）** | |
| --- | --- | --- | --- | --- | --- | --- | --- |
|  |  |  | **Low** | **High** |  | **Low** | **High** |
| **Age** | 1.64 | | 1.49 | 1.83 | 0.61 | 0.55 | 0.67 |
| **Gender** | 1.13 | | 1.07 | 1.27 | 0.88 | 0.79 | 0.94 |
| **Disease history** | 1.17 | | 1.09 | 1.30 | 0.86 | 0.77 | 0.91 |
| **Location** | 2.35 | | 2.10 | 2.66 | 0.43 | 0.38 | 0.48 |
| **Headache Nature** | 2.26 | | 2.03 | 2.55 | 0.44 | 0.39 | 0.49 |
| **Mood** | 1.20 | 1.12 | | 1.34 | 0.83 | 0.75 | 0.89 |
| **Fatigue** | 1.29 | 1.19 | | 1.43 | 0.77 | 0.70 | 0.84 |
| **Influenza** | 1.10 | 1.04 | | 1.24 | 0.91 | 0.81 | 0.96 |
| **Blurred vision** | 1.07 | 1.02 | | 1.23 | 0.93 | 0.81 | 0.98 |
| **Dizziness** | 1.09 | 1.03 | | 1.24 | 0.92 | 0.81 | 0.97 |
| **Photophobia** | 1.87 | 1.69 | | 2.10 | 0.53 | 0.48 | 0.59 |
| **Phonophobia** | 1.87 | 1.69 | | 2.10 | 0.54 | 0.48 | 0.59 |
| **Nausea** | 2.02 | 1.82 | | 2.27 | 0.49 | 0.44 | 0.55 |
| **Vomiting** | 1.89 | 1.70 | | 2.12 | 0.53 | 0.47 | 0.59 |
| **Hypertension** | 1.27 | 1.18 | | 1.41 | 0.79 | 0.71 | 0.85 |
| **Diabetes** | 1.16 | 1.08 | | 1.29 | 0.87 | 0.78 | 0.92 |
| **PFO** | 1.24 | | 1.15 | 1.38 | 0.81 | 0.72 | 0.87 |
| **Anxiety/Depression** | 1.07 | | 1.02 | 1.23 | 0.93 | 0.81 | 0.98 |
| **Cerebrovascular** | 1.22 | | 1.13 | 1.35 | 0.82 | 0.74 | 0.88 |
| **Menstruation** | 1.05 | | 1.01 | 1.25 | 0.95 | 0.80 | 0.98 |

PFO: patent foramen ovale; VIF: variance inflation factor.
